# HIV-Associated Dementia: Associated Factors and Characteristics of Cognitive Domain Abnormalities in Elderly People Living with HIV Treated with Highly Active Antiretroviral Therapy

**DOI:** 10.4269/ajtmh.22-0234

**Published:** 2022-10-31

**Authors:** Lakkana Boonyagars, Nucharee Kiatsoongsong, Supharat Winitprichagul

**Affiliations:** ^1^ Division of Infectious Diseases, Faculty of Medicine, Navamindradhiraj University, Bangkok, Thailand;; ^2^ Department of Internal Medicine, Faculty of Medicine, Navamindradhiraj University, Bangkok, Thailand;; ^3^ Division of Neurology, Faculty of Medicine, Navamindradhiraj University, Bangkok, Thailand

## Abstract

This study aimed to evaluate the prevalence and associated factors of HIV-associated dementia (HAD) in people living with HIV (PLWH) aged ≥ 60 years who are currently treated with highly active antiretroviral therapy. A cross-sectional study was conducted on adult (age ≥ 60 years) PLWH at the infectious clinic, Vajira Hospital, Navamindradhiraj University, Thailand, between August 2019 and March 2021. We collected the patients’ characteristics and performed Montreal Cognitive Assessment and Instrumental Activities of Daily Living test to determine whether they have HIV-associated neurocognitive disorders (HAND), which we further classified into asymptomatic neurocognitive impairment (ANI), mild neurocognitive disorder (MND), and HAD. Finally, we evaluated the prevalence, associated factors, and characteristics of cognitive domain abnormalities in these patients. We enrolled 84 elderly PLWH patients consisting of 43 (51.2%) males. The mean patient age was 63 years (SD ± 3.9), and the median duration of HIV infection was 13 (SD ± 5.7) years. All the patients had undetectable HIV viral load. Among them, seven (8.3%) had no neurocognitive impairment, 61 (72.6%) had ANI, three (3.6%) had MND, and 13 (15.5%) had HAD. After confounder adjustment, the patient age of ≥ 65 years was found to be significantly associated with dementia (odds ratio = 5.97, 95% CI: 1.51–23.57). Significant difference in the mean score of all cognitive domains was observed between the patients with HAD and those with normal cognitive status. HAND is common in PLWH. Age older than ≥ 65 years is a risk factor of HAD.

## INTRODUCTION

Neurocognitive impairment (NCI) is common among people living with HIV (PLWH), and this condition is currently referred to as HIV-associated neurocognitive disorder (HAND).^1^ HIV causes neuropathological changes in the basal ganglia and white matter, leading to subcortical dementia with deficits in memory, concentration, attention, and executive functions.^2^^,^^3^ The terminology of HAND was defined in 2007.^1^ According to the Frascati criteria, this syndrome is categorized into the following three stages by order of increasing severity: asymptomatic neurocognitive impairment (ANI), mild neurocognitive disorder (MND), and HIV-associated dementia (HAD).

After the introduction of highly active antiretroviral therapy (HAART), the prevalence of HAD, the severe form of HAND, has significantly declined.^2^^,^^4^^,^^5^ However, the incidence of less severe stages including ANI and MND persists at 20% to 50% and may occur early during HIV infection.^2^^,^^4^^–^^8^ The prevalence of HAND varies between 21% and 86%.^9^^,^^10^ Several risk factors associated with this disease are found in different demographics; clinical data such as age, sex distribution, education, country, stage of HIV disease, current/nadir CD4 cell count (especially ≤ 200 cells/mm^3^), early decrease in CD4 count, high initial plasma HIV viremia, HAART type, HBV/HCV coinfection, and low hemoglobin concentration are some of the factors associated with NCI in PLWH.^9^^,^^11^^–^^15^

NCI develops with advanced age.^16^ Given their long life expectancy, the high prevalence of HAND in people living with HIV is expected. Matched studies reported that, after the adjustment for major confounders including age, HIV infection was found to be significantly associated with an increased risk of NCI compared with that in HIV-uninfected individuals.^17^ This finding suggests that the pathogenesis of NCI in PLWH involves age-related and virus-independent diseases.^18^^–^^21^

Previous studies and meta-analyses lack data on the prevalence of HAND in the older age group because they only included a small proportion of patients older than 60 years.^5^^,^^15^^,^^21^^,^^22^ This study aimed to evaluate the prevalence and associated factors of HAND in PLWH aged ≥ 60 years who are currently treated with HAART.

## MATERIALS AND METHODS

We performed a cross-sectional study of adults (age ≥ 60 years) whose final diagnosis was HIV infection and are currently treated with HAART at Vajira Hospital, Navamindradhiraj University, between August 1, 2019, and March 31, 2021. Vajira Hospital is a 900-bed university hospital in Bangkok, Thailand, with more than 700,000 outpatient visits and approximately 30,000 inpatient admissions annually. Patients with communication problems and cannot undergo Montreal Cognitive Assessment (MoCA) and those who refused to participate were excluded. The patients’ history and clinical characteristics were obtained from the electronic database. This study was approved by the Vajira Institutional Review Board (COA 085/2562).

### Study measures.

#### MoCA test.

All participants were evaluated with MoCA and Lawton and Brody’s Instrumental Activities of Daily Living (IADL) scale by qualified personnel for neurocognitive function assessment.^21^^–^^24^ MoCA is well established in Alzheimer’s disease and has been used in the diagnosis of HAND.^24^^–^^26^ This test is suitable in the clinical setting because it can be administered in a short time (∼10–15 minutes) and is relatively comprehensive because it can evaluate multiple cognitive domains, such as visuospatial, naming, attention, language, abstraction, delayed recall, and orientation to time and place.^3^^,^^27^ The score ranges between 0 and 30, where ≥ 25 is considered normal and < 25 indicates cognitive impairment. For the participants with < 6 years of schooling, one point was added to their total score.^28^

#### The IADL scale.

The IADL scale is used to evaluate functional condition and is primarily designed to assess an individual’s ability to live independently across eight domains: the ability to use a telephone, shopping, food preparation, housekeeping, laundry, mode of transportation, responsibility for own medication, and ability to handle finances. The participants were scored by selecting which item was the most similar to their highest functional status (e.g., 0 or 1), and the summary score ranged from 0 (low function, dependent) to 8 (high function, independent) for women and 0 through 5 for men to avoid potential gender bias.^23^

MoCA and IADL tests are complementary; the former examines several cognitive areas, and the latter provides a functional evaluation. HAND was categorized according to the combined MoCA and IADL scores. IADL was required to differentiate ANI, MND, and HAD. All patients diagnosed with HAD/MND were referred to a neurologist and/or psychiatrist to exclude the other causes of cognitive impairment, such as Alzheimer’s disease, vascular dementia, and reversible cause of dementia by clinical diagnostic criteria and investigation as appropriate in each case. Frascati criteria were used to determine the HAND classification according to the MoCA and IADL scores.^1^

#### NCI diagnosis.

We use HAND criteria (known as Frascati criteria) developed in 2007 by a working group formed by the U.S. National Institute of Mental Health and National Institute of Neurological Diseases and Stroke.^1^ For cognitive screening, we used MoCA and Lawton IADL scale to evaluate the performance of the patients. MoCA test has been used in PLWH to evaluate HAND.^26^^,^^29^ The Thai version of the MoCA was translated by S. Hemrungrojn and back-translated by a linguistic staff person at the Chulalongkorn Language Institute.^30^^,^^31^ Thai-MoCA is a reliable and valid screening tool for NCI diagnosis in the Thai population^32^ and can discriminate patients with NCI from healthy controls with an area under the receiver operating curve of 0.813 and best cutoff scores of ≤ 24.^32^ Many studies used Thai-MoCA in different Thai populations, including in patients with schizophrenia,^30^ Alzheimer’s disease,^32^ preoperative geriatric patients,^33^ and PLWH.^34^

### Central nervous system penetration effectiveness score.

Central nervous system (CNS) penetration effectiveness (CPE) score is based on the pharmacokinetics and pharmacodynamics of each prescribed HAART and reflects each drug’s capacity to penetrate the CNS. A high CPE score is associated with a decrease in CSF HIV viral load and improved HIV virological control in the CNS.^35^ CPE was calculated for each HAART drug, and the scored ranged between 0 (absence of CNS penetration) and 4 (highest possible CNS penetration).^36^^,^^37^ The CPE score was calculated as the sum of the CPE scores of all drugs included in the HAART regimen, which was then categorized as having low (< 7) or high (≥ 7) CPE.^38^ The CPE scores are shown in Supplemental Table 1.

### Statistical analysis.

Variability in the prevalence of HAD was observed between studies. A systematic review of 26 studies of PLWH across eight countries revealed that prevalence ranged from 2.2% to 52% based on different diagnostic criteria.^39^ With 140 PLWH aged ≥ 60 years in our cohort, assuming the proportion of PLWH having HAD was 15% with 95% confidence and a margin of error of 5%, a total sample size of at least 82 was needed for the study.^4^^,^^40^^,^^41^ Categorical variables were presented as number (*n*) and percentage (%), and numerical variables were presented as mean and standard deviation (mean ± SD) or median with interquartile range (IQR) depending on their normality. Chi-square or Fisher’s exact test was performed for the univariable analysis of categorical data to identify the dementia-associated factors among PLWH. One-way analysis of variance or Kruskal–Wallis test were conducted depending on the variable’s normality to compare more than two groups of numerical variables (normal, ANI+MND, and HAD). Variables with a *P* value < 0.1 in the univariable analysis were included in a multivariable model. Multivariable logistic regression was performed using a backward stepwise elimination approach to construct the final model. Data analysis was conducted using SPSS version 22.0 (SPSS Inc., Chicago, IL). A *P* value < 0.05 was statistically significant in all analyses.

## RESULTS

**Figure 1.**
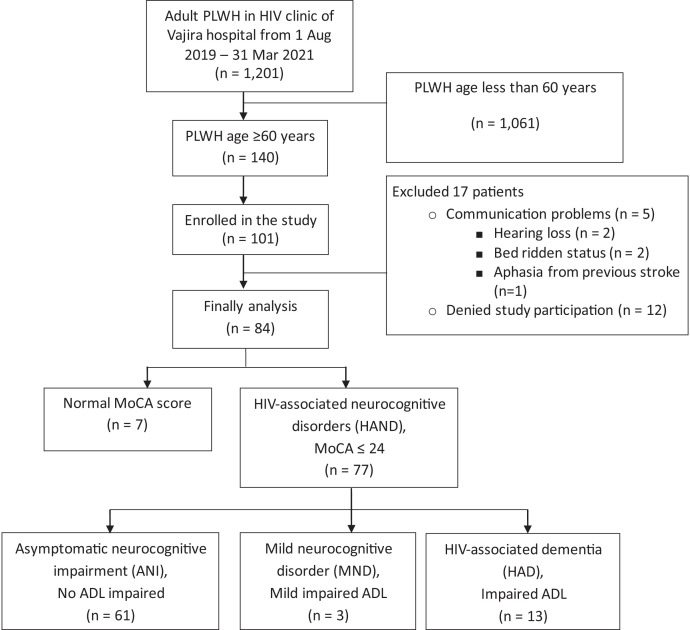
Study flow.

A total of 140 patients aged ≥ 60 years who was currently on HAART were followed up regularly at the infectious disease clinic of Vajira Hospital from August 1, 2019, to March 31, 2021. Among these patients, 101 were screened for study eligibility and enrollment, and 17 were excluded from the study (12 declined study participation, and five were unable to perform the MoCA test). Finally, 84 patients were included in the study. The study flow is provided in Figure 1.

Seven patients had normal MoCA scores. The remaining 77 patients had abnormal MoCA scores and were diagnosed with HAND. The overall prevalence of HAND was 77 out of 84 (91.7%) patients. These 77 patients were further classified as ANI (61, 79.2%), MND (3, 3.9%), and HAD (13, 16.9%).

The patients’ characteristics are provided in Table 1. The mean age of our patient cohort was 63.24 (SD ± 3.88) years, and equal numbers of males and females were enrolled (*n* = 42 each). The mean BMI was 22.8 (SD ± 4.93) kg/m^2^, and low body mass index (BMI; < 18 kg/m^2^) was found in 19 patients (22.6%). Sixty-two (73.8%) patients had comorbid disease, the most common of which were dyslipidemia (*n* = 50; 59.5%), hypertension (*n* = 33; 39.3%), and diabetes mellitus (*n* = 17; 20.2%). Two patients had a history of a remote cerebrovascular event with full recovery, two patients had well-controlled schizophrenia and had been followed regularly with a psychiatrist. None has HAD. No major depressive disorder or active substance use disorder were found in our patient cohort. The mean duration of HIV infection was 13.5 (SD ± 5.58) years, and the mean duration of HAART application was 10.6 (SD ± 4.8) years. Forty-one (48.8%) patients had HIV duration of ≥ 15 years. The median CD4 count and percentage before HAART were 187 (IQR: 47.25–323) cells/mm^3^ and 9.5% (IQR: 4–16), respectively, and those at the time of enrollment were 507 (IQR: 319.25–706.25) cells/mm^3^ and 24% (IQR: 18–30), respectively. All the patients had undetectable HIV viral load and exhibited good HAART adherence. The mean duration of undetectable HIV viral load was 8.7 (SD ± 3.49) years.

**Table 1:** Patient characteristics (*N* = 84)

Characteristics	*n* (%)
Age (years), mean (SD)	63.24 (3.88)
Age ≥ 65 years, *n* (%)	26 (31)
Male sex, *n* (%)	42 (50)
BMI (kg/m^2^), mean (SD)	22.8 (4.93)
BMI ≤ 18 kg/m^2^, *n* (%)	19 (22.6)
Year of education, median (IQR)	7 (4–12)
< 9 years, *n* (%)	46 (54.8)
Underlying diseases	77 (91.7)
Dyslipidemia, *n* (%)	50 (59.5)
Hypertension, *n* (%)	33 (39.3)
Diabetes mellitus, *n* (%)	17 (20.2)
Cancer, *n* (%)	4 (4.8)
Chronic kidney disease, *n* (%)	4 (4.8)
Other,* *n* (%)	28 (33.3)
Hepatitis profile (*N* = 79)	
Hepatitis B coinfection, *n* (%)	3 (3.8)
Hepatitis C coinfection, *n* (%)	6 (7.1)
Duration of HIV infection (years), mean (SD)	13.5 (5.58)
< 10 years, *n* (%)	19 (22.6)
10–20 years, *n* (%)	59 (70.2)
> 20 years, *n* (%)	6 (7.1)
CD4 count before ART initiation (cells/mm^3^), median (IQR)	187.5 (47.25–323)
Percentage CD4 count before ART initiation, median (IQR)	9.5 (4–16)
CD4 count at present (cells/mm^3^), median (IQR)	507 (319.25–706.25)
Percentage CD4 count at present, median (IQR)	24 (18–30)
Undetectable HIV viral load, *n* (%)	84 (100)
Duration of ART treatment (years), mean (SD)	10.61 (4.79)
Regimens of ART, *n* (%)	
NNRTI-based regimens, *n* (%)	63 (75)
PI-based regimens, *n* (%)	16 (19)
NNRTI + PI-based regimens, *n* (%)	6 (7.1)
CPE score, mean (SD)	7.20 (1.84)
≤ 7, *n* (%)	18 (21.4)
History of CNS infection, *n* (%)†	4 (4.76)
History of syphilis infection, *n* (%)‡	9 (10.7)
Adherence	
91%–100%, *n* (%)	73 (86.9)
81%–90%, *n* (%)	11 (13.1)

ART = antiretroviral treatment; BMI = body mass index; CNS = central nervous system; CPE = central nervous system penetration effectiveness; IQR = interquartile range; NNRTI = nonnucleoside reverse transcriptase inhibitor; PI = protease inhibitor.

* Other: chronic kidney disease, cancer, *n* = 4; hypothyroid, gout, asthma, psoriasis, coronary arterial disease, stroke, schizophrenia, *n* = 2; rheumatoid arthritis, pituitary adenoma, immune thrombocytopenia, chronic osteomyelitis, benign prostatic hyperplasia, allergic rhinitis, *n* = 1.

‡ 3 = cryptococcal meningitis; 1 = tuberculous meningitis.

‡ 3 = primary syphilis; 1 = secondary syphilis; 5 = late latent syphilis.

Regarding years of education, median total years was 7 (IQR: 4–12) years. Forty-six patients had finished sixth grade, 10 (11.9%) had finished 9th grade, and sixteen (19%) finished 12th grade. Twelve (14.3%) patients had a total education of more than 12 years.

With regard to HAART regimen, 63 (75%) patients using nonnucleoside reverse transcriptase inhibitor (NNRTI)-based regimens such as rilpivirine (*n* = 10, 15.9%), nevirapine (*n* = 25, 39.7%), and efavirenz (*n* = 28, 44.4%), and 16 (19%) patients used protease inhibitor (PI)-based regimens. The remaining patients (*n* = 5, 6%) received NNRTI plus PI regimens. No integrase inhibitors were applied. Among the 33 (39.3%) patients using three-drug fixed-dose combination in one pill, 11 received zidovudine/lamivudine/nevirapine and 22 were prescribed with tenofovir/emtricitabine/efavirenz. The mean CPE score was 7.20 (SD ± 1.84), which was divided into low (< 7) and high (≥ 7) scores for 18 (21.4%) and 66 (78.6%) patients, respectively. Four (4.76%) patients had a history of CNS infection (three = cryptococcal meningitis, one = tuberculous meningitis), and nine patients (10.7%) had a history of syphilis infection (three = primary syphilis, one = secondary syphilis, five = late latent syphilis). No history of neurosyphilis was detected.

The results of univariate analysis of dementia-associated factors in terms of neurocognitive status (normal versus ANI + MND versus HAD) are shown in Table 2. Significant differences in mean patient age were found among different neurocognitive statuses (*P* = 0.012). Patient age of ≥ 65 years was significantly associated with different neurocognitive statuses (*P* = 0.003). Significant differences in the median of the total year of education were observed among different neurocognitive statuses (*P* = 0.024). Total years of education < 9 years was significantly associated with different neurocognitive statuses (*P* = 0.032). Sex, BMI, underlying diseases, duration of HIV infection, CD4 cell count, HAART regimen, and efavirenz use were not associated with different neurocognitive statuses.

**Table 2:** Univariate analysis of the associated factors of dementia in terms of neurocognitive status (normal versus ANI+MND versus HAD)

Risk factor	Normal (*N* = 7)	ANI+MND (*N* = 64)	HAD (*N* = 13)	*P* value
Age (years), mean (SD)	63.6 (3.0)	62.6 (3.53)	66.1 (4.82)	0.012
Age ≥ 65 years, *n* (%)	3 (42.9)	14 (21.9)	9 (69.2)	0.003
Male sex, *n* (%)	4 (57.1)	33 (51.6)	5 (38.5)	0.64
Weight (kg), mean (SD)	67 (13.8)	60.3 (16.79)	52.6 (9.22)	0.13
BMI (kg/m^2^), mean (SD)	26.1 (3.12)	22.8 (5.23)	21.1 (3.22)	0.099
≤ 18 kg/m^2^, *n* (%)	0 (0)	16 (25)	3 (23.1)	0.32
Year of education (years), median (IQR)	9 (4–16)	7.5 (4–12)	4 (4–7)	0.024
< 9 years, *n* (%)	2 (28.6)	33 (51.6)	11 (84.6)	0.032
Underlying diseases, *n* (%)	5 (71.4)	46 (71.9)	11 (84.6)	0.628
Diabetes mellitus, *n* (%)	2 (28.6)	13 (20.3)	2 (15.4)	0.782
Hypertension, *n* (%)	3 (42.9)	24 (37.5)	6 (46.2)	0.827
Dyslipidemia, *n* (%)	5 (71.4)	38 (59.4)	7 (53.8)	0.746
Hepatitis B coinfection (*N* = 79), *n* (%)	1 (14.3)	6 (10)	0 (0)	0.468
Hepatitis C coinfection (*N* = 79), *n* (%)	0 (0)	5 (8.3)	1 (8.3)	0.729
History of syphilis infection, *n* (%)	1 (14.3)	6 (9.4)	2 (15.4)	0.775
History of CNS infection, *n* (%)	1 (14.3)	3 (4.7)	0 (0)	0.359
Duration of HIV infection (years), mean (SD)	11.7 (4.23)	13.7 (5.83)	13.77 ± 5.09	0.85
≥ 15 year, *n* (%)	2 (28.6)	31 (48.4)	8 (61.5)	0.369
First CD4 count (cells/mm^3^) (*N* = 76), median (IQR)	111 (39–243)	187 (45–328.75)	278 (79–423)	0.483
≥ 350 cells/mm^3^, *n* (%)	0 (0)	12 (20.7)	3 (27.3)	0.341
≤ 200 cells/mm^3^, *n* (%)	4 (57.1)	33 (56.9)	5 (45.5)	0.779
Last CD4 count (cells/mm^3^), median (IQR)	570 (253–651)	487 (343.25–665.75)	540 (305–825.5)	0.790
≥ 350 cells/mm^3^, *n* (%)	4 (57.1)	47 (73.4)	8 (61.5)	0.507
CPE score, mean (SD)	6.7 (1.89)	7.1 (1.88)	7.9 (1.50)	0.267
≤ 7, *n* (%)	6 (85.7)	39 (60.9)	6 (46.2)	0.224
ART regimen use, *n* (%)				
NNRTI-based regimen, *n* (%)	7 (100)	46 (71.9)	10 (76.9)	0.260
PI-based regimen, *n* (%)	0 (0)	13 (20.3)	3 (23.1)	0.396
Efavirenz use, *n* (%)	4 (57.1)	24 (37.5)	3 (23.1)	0.315
Tenofovir use, *n* (%)	5 (71.4)	37 (57.8)	6 (46.2)	0.539
Abacavir use, *n* (%)	1 (14.3)	10 (15.6)	2 (15.4)	0.996

ANI = asymptomatic neurocognitive impairment; ART = antiretroviral treatment; BMI = body mass index; CNS = central nervous system; CPE; CNS penetration effectiveness; HAD = HIV-associated dementia; IQR = interquartile range; MND; mild neurocognitive disorder; NNRTI = nonnucleoside reverse transcriptase inhibitor; PI = protease inhibitor.

In terms of CPE score, no significant association with different neurocognitive statuses was observed for the mean CPE score and the number of patients with low CPE scores (< 7).

The results of the multivariate analysis of dementia-associated factors in terms of neurocognitive status (ANI + MND versus HAD) are shown in Table 3. Patient age of ≥ 65 years was independently associated with dementia compared with patient age of < 65 years (adjusted odds ratio [OR] = 5.97, 95% CI: 1.51–23.57: *P* = 0.011). However, total year of education of < 9 years was not independently associated with dementia compared with total year of education of ≥9 years (adjusted OR = 2.97, 95% CI: 0.55–16.04, *P* = 0.206).

**Table 3:** Multivariate analysis of the associated factors of dementia in terms of neurocognitive status (ANI+MND versus HAD)

Risk factor	ANI + MND (*N* = 64)	HAD (*N* = 13)	OR (crude)	OR (adjusted)	OR adjusted (95% CI)	*P* value
Age ≥ 65 years, *n* (%)	14 (21.9)	9 (69.2)	8.036*	5.97	1.51–23.57	0.011
Year of education < 9 years, *n* (%)	33 (51.6)	11 (84.6)	5.167†	2.97	0.55–16.04	0.206

ANI = asymptomatic neurocognitive impairment; HAD = HIV-associated dementia; MND = mild neurocognitive disorder; OR = odds ratio.

* *P* = 0.002.

† *P* = 0.034.

**Figure 2.**
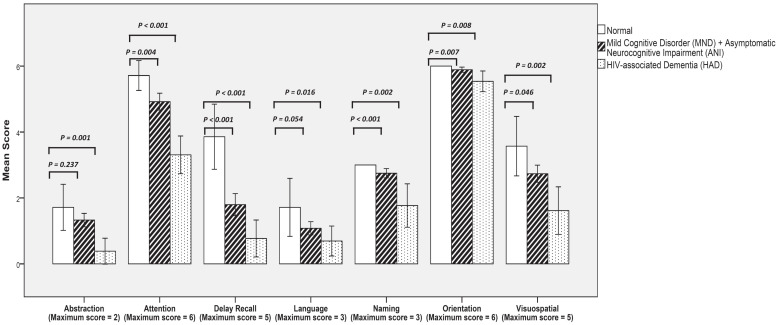
The mean and 95%CI of the Montreal cognitive assessment (MoCA) score in 7 domains.

The mean and 95% CI of the MoCA score in different domains for all the patients are shown in Figure 2. The seven domains were abstraction (maximum score = 2), attention (maximum score = 6), delay recall (maximum score = 5), language (maximum score = 3), naming (maximum score = 3), orientation (maximum score = 6), visuospatial (maximum score = 5). Significant difference in the mean scores in all seven domains was found between the patients with dementia and those with normal cognitive status (*P* ≤ 0.001 to 0.016). Significant difference in the mean scores in attention, delay recall, naming, orientation, and visuospatial domains was obtained between the patients with MND + ANI and those with normal cognitive status (*P* ≤ 0.001 to 0.007). No difference in the mean score in abstraction and language was observed between the patients with MND + ANI and those with normal cognitive status.

## DISCUSSION

This cross-sectional study involved 84 adult PLWH (aged ≥ 60 years) who were being treated with HAART. The prevalence of HAND in our study was high (91.7%). Among the 77 patients with HAND, 61 (79.2%) were asymptomatic. Only 13 (16.9%) patients were diagnosed with dementia. Overall, the prevalence of dementia in PLWH is higher than that in the normal population. In a matched study of aging individuals, HIV infection was significantly associated with an increased risk of NCI.^17^ The proposed mechanism of dementia in HIV is as follows: upon entering the CNS, HIV invades microglia and macrophages in the CNS, leading to chronic immune activation and macrophage dysregulation. The overproduction of proinflammatory cytokines results in neuronal damage and loss.^42^^,^^43^ With regard to the prevalence of HAND, Wei et al. conducted a systematic review of 18 studies and estimated the prevalence of HAND, ANI, MND, and HAD at 44.9%, 26.2%, 8.5%, and 2.1%, respectively.^15^ The relatively high prevalence of HAND in our study could be explained by the high average patient age in our cohort; the mean age was 63.24 years, and more than 60% of our cohort have an age of more than 65 years. A recent study from Mexico included 206 participants on HAART with a mean age of 60.5 years and reported the prevalence of HAND at 66.0%.^44^ In addition to age, several factors might explain the high prevalence of HAND in our study, including the nadir CD4 cell count and education level. In several studies, nadir CD4 cell count was significantly associated with the NCI in PLWH.^4^^,^^5^^,^^41^ It reflects the level of immunosuppression that cause irreversible CNS injury before HAART treatment (so-called legacy effect).^45^^,^^46^ In our study, the median nadir CD4 cell count was only 187.45 cells/mm^3^, considerably lower compared with studies from other countries.^47^ A study from France revealed a lower prevalence of NCI, which was 58.5% with a higher median (IQR) nadir CD4 cell count of 260 (154–385) cells/mm^3^.^48^ Concerning the education level, lower educational level has been associated with higher prevalence of mild cognitive impairment.^49^ The median total education year in our study was only 7 years. This could be one explanation related to the high prevalence of mild cognitive impairment in our study.

Many factors are associated with HAND. Regarding old age, the odds of PLWH developing HAND increases with age, similar to dementia in people living without HIV.^18^^,^^20^ Our study found that age ≥ 65 years was independently associated with dementia (adjusted OR = 5.97) among PLWH. A recent systematic review by Wei et al. reported the following factors that are significantly associated with HAND: female sex, low education level (below college), low current CD4 cell count < 500 cells/mm^3^, and study being conducted in underdeveloped countries (compared with developing and developed countries).^15^ Our research revealed that the total year of education < 9 years was associated with dementia among PLWH (with adjusted OR = 2.97, 95% CI: 0.55–16.04); however, no statistical significance was found in the multivariate analysis (*P* = 0.206). From the wide CI in our analysis, this finding could be explained by factors such as the small sample size and variation in the total education years of our cohort, which ranged vary from 2 to 21 years. Sex and current CD4 level were not associated with dementia. One explanation for this finding is the small sample size of our study.

In terms of CPE score, our study revealed that less than 25% of our patient cohort used HAART regimens with low CPE scores (< 7). We also found no association between HAART regimens with low CPE score and dementia. In theory, low scores indicate low CNS penetration. Some studies have shown that high CPE scores are associated with low CSF HIV viral loads.^35^^,^^36^ However, the supporting data on the impact of CPE and how low CSF HIV viral loads lead to neurocognitive improvement are unclear and inconsistent across studies. Two observational studies supported that HAART regimens with high CPE scores are correlated with a low prevalence of NCI.^50^^,^^51^ However, a randomized controlled clinical trial including 120 participants at five study sites in 2007 revealed that the improvement in neurocognitive performance was nonsignificant in the high CPE score arm compared with that in the control arm; the study was terminated early due to slow accrual and a low likelihood of detecting a difference in the primary outcome.^52^ A recent small prospective, open-label pilot randomized controlled trial enrolled 14 virological suppressed HAND patients revealed a global neurocognitive improvement in the combination HAART enhancement with maraviroc arm compared with that in the control arm without maraviroc over time.^53^ A large cohort study published in 2019 enrolled 981 virologically controlled PLWH reported no association between NCI and CPE score, whether cross-sectional or cumulative.^38^ Dolutegravir (DTG), a new integrase strand transfer inhibitor, is currently included in the preferred regimens in worldwide HIV treatment guidelines. Owing to the good CNS penetration and high CPE score of DTG, a high CPE score is expected for this HAART regimen.

Regarding MoCA scores, the predominant clinical feature of HAND is subcortical dementia with early findings of deficits in concentration, attention, and memory domains. Motor signs are less prominent.^3^^,^^54^ Although we found abnormality in multiple domains, the orientation domain is considered satisfactory. The mean score in all domains was significantly lower in patients with HAD compared with normal patients. Even in patients with mild cognitive impairment (ANI and MND), significantly lower mean scores in attention, delayed recall, naming, orientation, and visuospatial domains were still detected compared with those in patients with normal cognitive status. These findings are comparable to the study of Koenig et al., who found significantly different mean scores for several MoCA tests including those in the domains of naming, orientation, and delayed recall between the HAND and non-HAND groups.^29^

Our study has several strengths. First, the wide range of disease severity of cognitive impairment in our cohort reflects the efficiency of MoCA and IADL test to evaluate cognitive impairment in PLWH. Second, both tests were administered by the same physician team throughout the study period, thus making the precision in our study high and reliable. Finally, we used MoCA and IADL tests, two well-known methods that are acceptable for worldwide HAND diagnosis. Furthermore, the Thai MoCA test is well established and has a proof of validity comparable with the original MoCA test. This test has standard references already published in Thai version.

Our study also has several limitations. First, the small patient cohort might have resulted in underpowering and bias introduction. A large study population might have yielded the association of HAD with previously reported risk factors including sex and current CD4 cell count. The next limitation is that the majority of HAART regimens used in our study are based on NNRTI, which is not preferred for the present HIV treatment guidelines. This research was conducted before the implementation of integrase inhibitors in the preferred Thailand guideline regimens (DTG was introduced in the Thailand HIV treatment guideline in 2020). Owing to the inconclusive data on the association between HAND and CPE score, this limitation is somehow less concerning.

This study is the first to evaluate NCI in Thai elderly PLWH. The prevalence of HAND is high, particularly in less severe forms (ANI and MND). Old age (≥ 65 years) was the risk factor of HAD in patients currently treated with HAART. Significant difference in the mean score of all seven domains (abstraction, attention, delay recall, language, naming, orientation, and visuospatial) was observed between the patients with HAD and those with normal cognitive status. Prospective data collection with high patient enrollment rate and additional investigations including neuroimaging could provide additional information on the clinical course and pathogenesis of HAND.

## Supplemental files


Supplemental materials
