# Supplementary material for: HIV-Associated Dementia: Associated Factors and Characteristics of Cognitive Domain Abnormalities in Elderly People Living with HIV Treated with Highly Active Antiretroviral Therapy
Source: Am J Trop Med Hyg. 2022 Oct 31;107(6):1250–7. doi: 10.4269/ajtmh.22-0234 (PMC9768268; doi:10.4269/ajtmh.22-0234)
Supplement: Supplementary file 1 [file tpmd220234.SD1.pdf]

Supplementary Table 1. Central nervous system Penetration Effectiveness (CPE) ranking score

Central nervous system Penetration Effectiveness ranking score				
Drug class	4	3	2	1
Nucleoside reverse transcriptase inhibitors	Zidovudine	Abacavir<br>Emtricitabine	Didanosine<br>Lamivudine<br>Stavudine	Tenofovir<br>Zalcitabine
Non-nucleoside reverse transcriptase inhibitors	Nevirapine	Delavirdine<br>Efavirenz	Etravirine	
Protease inhibitors	Indinavir/r	Darunavir/r<br>Fosamprenavir/r<br>Indinavir<br>Lopinavir/r	Atazanavir<br>Atazanavir/r<br>Fosamprenavir	Nelfinavir<br>Ritonavir<br>Saquinavir<br>Saquinavir/r<br>Tipranavir/r
Entry/Fusion inhibitors		Maraviroc		Enfuvirtide
Integrase strand transfer Inhibitors	Dolutegravir	Raltegravir		

Abbreviations: /r; ritonavir.
